# Shared decision making - a review of its evaluation, efficacy, and applicability in asthma

**DOI:** 10.3389/fmed.2025.1639805

**Published:** 2025-08-12

**Authors:** Adrianna Piątkowska, Kamil Marszałek, Natalia Krupińska, Elizabeth Malaya, Magdalena Adamczewska, Piotr Kuna, Michał Panek

**Affiliations:** Department of Internal Medicine, Asthma and Allergy, Medical University of Lodz, Lodz, Poland

**Keywords:** asthma, patient-centered, communication, shared-decision making, patient-physician partnership

## Abstract

Over the years, the approach to medical decision-making has evolved significantly–from the traditional paternalistic model, in which decisions were made on behalf of the patient, to Shared Decision Making (SDM), which actively involves patients in the process. Given that a strong patient–healthcare provider relationship is a key factor in effective treatment, the literature increasingly highlights the importance of incorporating patient preferences. To achieve this, patients must receive clear explanations about their condition and treatment options, as well as care plans tailored to their individual needs. This is particularly relevant in conditions requiring long-term treatment, where outcomes depend heavily on patient adherence, motivation, and consistency. Chronic diseases like asthma require ongoing cooperation and trust between patients and healthcare providers. Asthma, one of the most common chronic respiratory conditions, has no curative treatment; its management relies on daily inhaled medications to control symptoms and prevent exacerbations. Several models have been developed to structure SDM implementation, ranging from basic frameworks promoting engagement to comprehensive approaches emphasizing environmental readiness and professional education. Key components include blended learning for healthcare providers and standardized tools to operationalize SDM, such as Patient Decision Aids (e.g., Written Asthma Action Plans) and multilingual resources like “Asthma Australia.” These tools are particularly valuable in overcoming barriers such as language and cultural differences, which can hinder access to care–especially for minority groups. Discrimination and lack of tailored communication can disproportionately affect patients with intellectual disabilities and those from culturally diverse backgrounds, highlighting the need for inclusive, individualized approaches in SDM-based chronic disease care. Although SDM shows promise in improving patient satisfaction, adherence, and potentially reducing healthcare costs, robust evidence remains limited. Global studies are needed to assess its true efficacy across various chronic conditions. This review aims to systematically analyze SDM models described in the literature, align them with asthma management requirements, incorporate patient needs and expectations, and propose practical strategies for integrating SDM in asthma care and other chronic respiratory diseases. Trust in healthcare providers is associated with improved health outcomes and increased treatment adherence, like for example “Asthma Australia” barriers to satisfactory care remain, particularly for patients from minority groups, who often face linguistic challenges, The structured integration of Shared Decision-Making (SDM). Although SDM has the potential to enhance patient satisfaction, improve adherence, and reduce healthcare costs is promising, conclusive evidence remains limited. The potential gain is not however excluded. There is a need for more data from studies on global scale to objectively determine its efficacy and applicability in various chronic conditions.

## 1 Introduction

Patient-centered care has evolved over the years, resulting in the shift from the idea of paternalism having healthcare providers (HCPs) completely responsible for decision making, to the more cooperative one of including the patients in this process according to their expectations to the (1). This modern idea has established the term prevailing in medical literature known as Shared Decision Making (SDM). SDM describes a model of diagnosis and treatment based on such collaboration involving both the patient and the HCP (2). From the patient’s perspective, it’s beneficial as it can improve feelings of autonomy, competence, and relatedness (3). The correlation with adherence however differs between studies and its impact in this area remains with limited evidence (3–6). Nonetheless, implementation of SDM methods during consultations have been reported to raise the probability of proper treatment choice (7), as well as to improve patients’ satisfaction as a cost and time effective method (8).

Asthma is a chronic respiratory disease without the curative treatment and with the disease management relying on symptom control and exacerbation prevention facilitated by MART/SMART (Maintenance and Reliever Therapy respectively without and with single inhaler) inhalative treatment scheme (9). MART/SMART therapy requires consistency and regular administration of the medication (9). The consistency of regular inhalative medication intake, is important According to the Global Initiative for Asthma (9), developing a good partnership between patients and physicians is among the significant factors contributing to asthma management as individual preferences and experiences vary (9). Asthma often restricts daily activities, affects social interactions, and requires lifestyle adjustments. It is a condition burdened with fear of exacerbations, uncertainty regarding medication effectiveness, difficulties obtaining inhalers and negative interactions with HCPs. All these factors present barriers to effective treatment. There is no universal and optimal approach to treat asthma, as individual experiences and preferences vary (GINA) (9). SDM may aim at some of these individual factors. The guidelines recommend applying SDM methods in the choice of the inhaler (GINA) (9) and more tools are in development to facilitate patients decision making e.g., Written Asthma Action Plans (WAAPs) (10). Yet the literature emphasizes a few more areas of interest to which SDM could be implemented. For instance, the frequency of medication intake, onset of action, efficacy in symptom control, inhaler technologies (e.g., built-in dose counters), access to healthcare facilities and maintenance of physical activity (especially in pediatric patients that feel limited by asthma symptoms) (11, 12).

Several models have been developed to facilitate the structured implementation of SDM, ranging from basic frameworks focused on enhancing patient involvement to more advanced models highlighting the importance of environmental preparedness and professional training. Core components underpinning these models include professional education (utilizing blended learning approaches that integrate in-person and online methods) and standardized instruments that enable SDM in clinical settings, such as Patient Decision Aids and multilingual materials. These resources are highly valuable since barriers to adequate care persist, especially due to language difficulties experienced by minority populations. Despite numerous models with promising general health outcomes (such as treatment efficacy, quality of life, adherence, patient-centered care) there is a need for more data and research conducted on a global scale, to better determine SDM’s efficacy and applicability. Advances in psychology, social science, and technology have become deeply integrated into modern family medicine, enhancing both prevention and treatment. Emerging concepts like complex adaptive systems offer a powerful framework for understanding health as a dynamic, context-dependent process. Together, these developments support a more holistic, personalized, and systems-oriented approach to patient care (13). This review aims to systematically analyze the current knowledge on shared decision-making (SDM) approaches described in the literature, align them with the requirements of asthma treatment and management, incorporate patients’ preferences and needs, and ultimately propose strategies for implementing SDM models in asthma care and potentially in other chronic respiratory diseases. The analysis focuses on studies addressing both challenges and potential solutions relevant to asthma, using search terms such as asthma, shared decision-making, barriers, and implementation. Based on the most recent literature (published within the past 5 years), this review presents current evidence on the barriers faced by individuals with asthma, available solutions, and the application of SDM models in delivering adequate care. In addition, a conceptual model is proposed, outlining key components of the clinical encounter that may contribute to building patient trust and facilitating effective implementation of SDM. This review aims to systematically analyze the current knowledge on shared decision-making (SDM) approaches described in the literature, align them with the requirements of asthma treatment and management, integrate patients’ preferences and needs, and ultimately develop and propose possible strategies for implementing SDM models in asthma therapy and potentially in other chronic respiratory disease this review addresses.

## 2 Patients vs. HCPs perspective on SDM

Both asthmatic patients and their healthcare providers encounter numerous challenges in achieving accurate diagnoses and developing optimal treatment plans, with the underlying issues often perceived differently due to contrasting perspectives. Barriers frequently identified in the literature include communication obstacles, unmet educational needs, the co-occurrence of psychiatric disorders, a lack of empathy and trust, poor adherence, and systemic factors within the healthcare system that undermine confidence in its purpose and effectiveness. Additional contributing factors encompass unreported or insufficiently reported symptoms in children, as well as sociodemographic determinants–such as low educational attainment, female sex, specific occupational exposures (in Colombia), older age, low income (in Australia), and limited language proficiency among immigrant children (14). HCPs, although the problems responsible for those obstacles may be perceived differently due to the contrast of their perspective. Among the barriers often described by literature are health care system-related factors that undermine the trust in its purpose and effectiveness (14). Contributing factors include unreported or insufficiently reported symptoms in children, and sociodemographic determinants such as low educational attainment, female sex, occupational exposures (Colombia), older age, low income (Australia), or limited language proficiency among immigrant children (US) (14).

The diagnostic inaccuracy might be one of the examples of the trust deterioration (15). That about 20%–73% of asthma cases are unrecognized, depending on the population studied (14). In some situations asthma does situate a challenge in establishing the proper diagnosis, as some symptoms may overlap with other conditions or simply be unspecific (e.g., cough). Several conditions, such as inducible laryngeal obstruction, chronic obstructive pulmonary disease (COPD), hyperventilation syndrome, and panic disorders, can present symptoms like asthma and contribute to diagnostic errors (16). In one study, 613 Canadian adults with a prior asthma diagnosis underwent a stepwise reassessment protocol involving spirometry, bronchial provocation testing, medication withdrawal, and specialist review. Asthma was ruled out in 33% of participants, and after 12 months, 30% remained off asthma medication (17). Misidentification of asthma may result in the delay in the identification and management of relevant conditions, poorly controlled symptoms, leading to frequent exacerbations and airway remodeling (15). Moreover, both under- and overdiagnosis do not only lead to inefficient disease management, they can also hinder the sole idea of SDM, as decisions based on incorrect assumptions may lead to inappropriate treatment or harm (15). Among other obstacles faced in asthma care is the frequent co-occurrence with psychiatric conditions. The literature reports that asthma patients T are more likely to experience mental disorders and use psychiatric medication than their peers without asthma, even when matched for age, gender, and geographic location. For instance, mental health disorders were identified in 15.1% of men and 32.9% of women with asthma, compared to 6.8% and 18.1% among their non-asthmatic counterparts, respectively. Similarly, psychiatric medication use was reported by 15.3% of men and 33.4% of women with asthma, versus 8.3% and 21.0% in the control group, respectively (18). Psychological distress significantly increased the risk of asthma exacerbations, manifesting as hospitalizations, unscheduled doctor visits, and emergency department visits. These effects were especially pronounced when psychological symptoms persisted beyond a year (19). While emotional distress was indicated to cause upcause to 10%–15% of asthma exacerbations. The proportion of patients experiencing acute flare-ups due to psychological stress increased with the disease’s severity (20). Moreover, anxiety related to heightened sensitivity to dyspnea could lead to more negative health perceptions (18). Among obstacles for implementation of SDM among asthma patients with coexisting mental disorders, literature indicates factors such as limited decision-making capacity, poor insight, or therapeutic pessimism on the part of healthcare professionals. Difficulties may also arise from personal dislike or conflicting recovery orientations and goals of care between the patient and professional (21).

Non-Hispanic Black or African American adults are 30% more likely to have asthma in comparison to non-Hispanic white Adults. Moreover, African Americans have an asthma-related mortality rate twice as high as White Americans (22). Ethnic minority groups represent a distinct population of patients with asthma who face unique challenges in the diagnostic process and disease management, largely due to cultural factors and language barriers (23). A limitation in care delivery is miscommunication, which is often exacerbated by limited English proficiency and the process of acculturation (24). For instance, African American patients may describe breathlessness with terms associated with the upper airway, rather than the chest or lower airway, potentially complicating accurate diagnosis, and treatment (25). The use of non-standard medication names has been associated with higher risk of additional corticosteroids, increased emergency department (ED) visits, and hospital admissions (26). Immigrant patients frequently encounter challenges in comprehending medical recommendations and may perceive a lack of clarity regarding their treatment plan (27). In a study conducted in Australia, many healthcare professionals (HCPs) reported that patients from culturally and linguistically diverse (CALD) backgrounds experienced difficulties with asthma self-management. A significant number of these patients were unfamiliar with the local healthcare system and tended to rely on personal beliefs rather than scientific evidence. Effective communication was also hindered by both language barriers and social isolation (28). Lower socioeconomic status, which often occurs alongside limited health literacy, is more common among those groups, contributing to higher rates of obesity, improper inhaler technique, and increased hospital admissions (29). Targeted, culturally tailored asthma management programs may help address these disparities. Culture-specific interventions have an impact on asthma courses. Implementation of those has improved asthma quality of life in two of seven studies in adults, while among children they were effective for usual care in reducing exacerbations requiring hospitalization and increasing asthma control in one study and enhancing patient’s quality of life in three studies (30).

89% of asthmatic patients have at least one unmet educational need. Common concerns among patients are the safety of the treatment and risk of exacerbations (31, 32). Additionally, patients with lower education levels have poorer understanding compared to those with university degrees (4). A recurring challenge patients face is the misunderstanding of correct inhaler use, often confusing rescue and control inhalers, which can lead to suboptimal asthma management (33). Nowadays, there is a vast variety of inhalators on the market, offering different ways of dosing or formulation. Patients who use more than one type of inhalator have worse outcomes, also, those who understand the need for inhalator are more likely to have better adherence (34). More complex treatment plans, in general, may have a negative impact on medication compliance. A study in Ethiopia showed that patients, who had lower or moderate complexity of medication regimens, had higher adherence and control over asthma (35). Moreover, modifications of medicine regimens and more complicated language result in higher risk of errors (36). According to patients, the most important factors when choosing the right inhaler were dose counter, feedback mechanism, impact on the environment, price, frequency of use, and being easy to use and carry (37). Implementation of SDM may be helpful, as it makes it more likely that the chosen inhaler suits the patient’s needs, and that they are educated on correct use. Additionally, those who deal with severe asthma want to understand how to manage the exacerbations and obtain information on treatment using monoclonal antibodies (31). While HCPs recognize patients’ need for education, they report feeling constrained by limited time during visits and insufficient patient education (38). Patients also acknowledge that education on treatment to medication may facilitate medication adherence (39), however, some report conflict and misinformation from their provider (11).

An important factor is that effective communication between HCPs and patients may be also hindered by a lack of empathy and trust. Empathy fosters the benefits of SDM by promoting patient autonomy and encouraging active participation in the treatment and self-management, strengthens the sense of partnership, helps de-escalate potential conflicts, and enables clinicians to demonstrate genuine interest in the patient’s condition (40, 41). One of the earliest studies exploring the influence of empathy on health outcomes was conducted in 2009, where patients with the common cold assessed their HCPs’ empathy using the Consultation and Relational Empathy (CARE) measure. Patients who gave their HCP highest scores experienced milder symptoms and shorter illness duration (40). The interconnection between SDM, team dynamics, and empathy has been shown to be significant in rehabilitation settings, where providers’ empathy levels were positively correlated with both patient satisfaction and treatment acceptance (42). Similarly, the Jefferson Empathy Scale (JSE) has been used to assess HPCs’ empathic ability. Higher empathy scores reported by asthmatic patients in JSE correlate with reduced patients’ anxiety, lower serum IL-6 levels, improved self-efficacy, and better sleep quality (43). Empathic communication enhances patient-centered care by helping individuals feel valued, supported, and respected (44). A meta analysis which included 55 studies, found that physician empathy is significantly associated with better health outcomes in oncology patients (45). Similar large scale studies are still needed in the context of asthma to confirm whether these benefits also apply to patients with chronic respiratory conditions. Perceiving providers as compassionate and understanding has been positively associated with overall trust, confidence in HCP’s benevolence and competence, and the quality of therapeutic relationship (46). Physicians who were able to listen to their patients with severe asthma with empathy were more likely to engage in shared decision-making, even with patients with whom the initial relationship had been difficult (47). Further empirical validation, including pre-test or pilot data, remains necessary to confirm its effectiveness in clinical settings.

Making process individual factors such as medication beliefs, including concerns about side effects and dependence, as well as preferences for complementary and alternative medicine (CAM), may influence adherence (24), which is a key component of treatment effectiveness. Therefore, these factors should be considered when selecting a management approach. Factors like female sex and uncontrolled asthma are significantly associated with more frequent CAM usage (48). The influence of CAM on patient adherence is not yet confirmed. Among families using such therapies, the use of non-conventional treatments was generally associated with reduced adherence to prescribed asthma management, with the exception of one ethnic group. Nonetheless, families who used CAM did not appear to view these therapies as a substitute for conventional treatment. In some cases, medication substitution may have been linked to barriers in accessing standard care or to concerns about potential side effects (49). In certain groups of patients who are skeptical of conventional treatments, the integration of CAM along with standard asthma medications could be proposed to enhance adherence. Children represent a distinct population in asthma management, with adherence patterns differing markedly from adults. The highest adherence rates were found in children prescribed montelukast alone while the lowest rates were found among children prescribed fluticasone alone. Younger child age was associated with higher medication adherence (50). Other studies had reported a mean adherence of 36% to inhaled corticosteroid among asthma children aged 5–16 (51). Parental beliefs about medication necessity and family organization are significant predictors of asthma adherence (50). Specific attitudes to parents’ perception of the disease influence medicine intake by their child and should be assessed during SDM (51). Adolescence is also associated with distinct barriers to achieving adequate adherence. This developmental period is characterized by a growing sense of independence, the formation of personal health beliefs, and an increasing reluctance to parents involvement in asthma management. Factors contributing to reduced adherence during adolescence include too time-consuming treatment regimens and lack of time, forgetfulness, embarrassment about using inhales in front of peers, ans the co-occurrence of depression and anxiety. According to a study (52), systemic factors may also influence the effectiveness of SDM. Household income is a significant determinant of asthma treatment. Continuity among Taiwanese patients, a positive association was observed between income level and willingness to pay for a HCP (53). Financial barriers among low-income families affected asthma management, with 9,3% of parents reporting reduced medication use for their children due to constant concerns. This highlights the need to tailor treatment plans to patients’ financial circumstances during SDM, for example, by selecting more affordable therapeutic options or incorporating telemedicineF resulted in using lessing (54). Finally, marital status has also been shown to affect asthma management. Being a single mother is associated with lower adherence during pregnancy. 30% of single pregnant women reported not taking medication regularly, and 39% either discontinued or reduced their asthma medications during pregnancy (55). Recognizing and addressing such diverse contextual and individual factors is critical for developing asthma treatment plans that are both effective and acceptable to patients, regardless of life stage or social context. Those key elements are summarized in Table 1.

**TABLE 1:** Perceiving of the treatment management by patient and HCP in the context of SDM approach.

Stance	Perspective	Example	Interventions	[Source]
Obstacles	HCP	Vague definition of SDM without clear objectives.	SDM trainings	(106)
		Lack of time	Compromise approach “everyday SDM” - with brief therapeutic recommendations from HCPs and supporting patient’s final decision	(107)
		Busy and noisy ward environments	Create a private place	(108)
		Low health literacy in patients	Less textual information, development of health literacy skills - functional, communicative and critical skills	(109)
	Patient	Stigma about asthma as a disease/taboo	Patient’s education and disease awareness	(110)
		Doubts about the accuracy of asthma diagnosis	Realization about episodic nature of asthma; tailored communication between HCP and patient	(111)
		Lack of knowledge of therapeutic options	Ensuring about all medical solutions	(108)
		Perceived lack of efficacy and fear of side effects	Fight with irrational or erroneous beliefs by proper explanation of treatment scheme	(111)
Preferences	HCP	Provide feedback	Assessment of the conversation between HCP and patient	(99)
		Provide private space	Ensuring no additional outside people during shared decisions	(108)
	Patient	Convenient treatment adjusted to patients’ preferences	The treatment in a form of inhalers should contain more doses, last longer and contain dose counter	(11)
		Positive relation with HCP	Patients want to be taken seriously, they want to be empowered by health professionals to manage their asthma.	(11)
		Development of strategies to feel control of asthma	Recognize specific asthma triggers, immediate access to inhalers	(11)
		Preference for written asthma action plan	Patients want to have MART approach in written form to better remember it	(75)

## 3 Known SDM models

The first structured description of SDM was introduced by Charles et al. (56). According to that, SDM needs at least two active participants, typically a patient and healthcare provider, who must both share information, build consensus, and ultimately reach an agreement on the treatment plan (56). This general model was evaluated in the context of asthma care in a 2009 study, which demonstrated that patients who experienced SDM during treatment showed better adherence to inhaled corticosteroids (ICS) and long-acting beta-agonists (LABAs), reduced use of short-acting beta-agonists (SABAs), and perceived a more equal role in decision-making compared to those receiving usual care or care based on a clinician-directed decision-making. Nevertheless, during the second year of observations adherence lowered in both groups showing that for prolonged impact of SDM, that was introduced mainly during the first time visit, there is a need for other interventions (6). Then, multiple models have been proposed, each of them taking a different approach to adjust to a variety of clinical situations.

One of the aims of SDM frameworks is to enhance patient-provider communication and cooperation. The Three Talk Model, for instance, presents SDM in three sequential steps: “choice talk,” “option talk” and “decision talk.” While straightforwardness makes it accessible for clinical use, it may oversimplify the complexity of interhuman interaction (57). In 2019 a study to evaluate efficacy of The Three Talk Model with simulated asthma patients was conducted. It consisted of two consultations, before and after SDM skills training based on the model. The OPTION5 scale was used to test perceived physician’s efforts to involve the patient in the process, and Shared Decision Making Questionnaire to measure, both physicians and patients, perspective on the process. Most physicians noticed they paid more attention to their communication, and more patient-centered approach, however, some felt the time pressure in outpatient care prevents them from asking open-questions (58). While showing promising results, the study was conducted on a small number of individuals, and only with simulated patients. More real-life research is needed.

Elements like cultural and contextual factors are highlighted in models such as MIND-IT, a conceptual framework, in which elements that interplay in that process are presented. It reminds clinicians to consider how their own and their patients’ beliefs, values, experiences, and cultural backgrounds interact during the decision-making process. Proper interpretation and understanding of those factors can facilitate consensus (59). 3-Circle Conceptual Model and Multistep Shared Decision-Making Pathway, highlight the importance of contextual influences, such as family involvement, social support, and patient’s illness history. The Multistep Pathway, created to implement that framework, underlines the value of explanation of these factors during medical encounters (60). Nethertheless, despite acknowledging the need to consider patients’ personal beliefs and values, these models do not provide in-depth guidance on how to adapt care to individual circumstances.

A key component of the Longitudinal SDM model is the use of Patients Decision Aids (PtDAs), that is also highlighted in MIND-IT model, but it expands this concept by recommending the use of educational materials even before clinical visits. In this approach, to ensure proper informed decision, patients should be provided with educational materials and review them, but also have preliminary discussion with decision partners, possibly consult other sources, and later clarify questions during consultation. Post-visit materials reinforce understanding and aid follow-up. This structure addresses patients’ educational needs regarding their condition and treatment options. Proper implementation of those in different steps of the decision-making process can help educate patients (59, 61). This model requires follow-up visits to ensure successful application. In Sweden only one in three patients had another visit during a 15-months period (62), in Korea 68,1% of patients were regular visitors (meaning having at least 3 visits per year) (63). In contrast, emerging evidence suggests that providing patients with relevant asthma-related information prior to the clinical encounter may actively support SDM. By enhancing patients’ knowledge, confidence, and sense of preparedness, such pre-visit interventions appear to foster greater awareness and engagement in the decision-making process. This effect has been reported as particularly beneficial among individuals from ethnic minority backgrounds, who may otherwise experience communication barriers or lower levels of participation. Nevertheless, due to the limited sample sizes in existing studies, further large-scale research is necessary to confirm these findings and explore their applicability across diverse populations (64).

Specialist consultations are another important aspect of SDM, especially in complex cases. The IP-SDM model (Interpersonal Shared Decision Making) emphasizes collaborative input from multiple HCPs through the process if needed (65). Creating a conductive clinical environment also supports effective SDM. The Four Habits Model focuses on communication skills that promote trust and empathy. It includes techniques such as adapting language and posture, building rapport early in the consultation, and recognizing both verbal and non-verbal emotional cues, such as facial expressions or silence, to convey empathy (66).

While these models vary in structure and focus, comprehensive frameworks such as the SHARE Model (2015) and the more recent 6-Steps of SDM Model (2023) integrate essential elements including patients’ engagement, explanation of treatment options, assessment of preferences, and collaborative decision-making (67, 68).

Based on The SHARE model another matrix was created. Purposeful SDM further categorizes SDM approaches according to clinical context. It highlights that SDM is not limited to choosing between treatment options but also includes aligning care plans with what matters most to the patient, which is particularly relevant in chronic care or life-threatening situations (69).

Most models of SDM still lack a thorough evaluation of efficacy and applicability. Although many of them acknowledge the importance of individual patient needs and the use of educational materials to support understanding, they often lack guidance on how to effectively tailor these interventions for populations with limited health literacy or restricted access to information.

For better comparison of those models and their pros and cons we have summarized them in Supplementary Table 1.

## 4 Promotion and implementation of SDM

Training of healthcare professionals is one of the critical factors for the successful implementation of SDM in clinical practice. Over the past decade, such training programmes have become increasingly diverse and are now targeted at various professional groups across different clinical contexts (70). The effectiveness of such training programs has shown a moderate impact on patient outcomes and a medium-large effect on observer-reported SDM skills. In terms of instructional format, blended approaches, combining online and live methods, have been found to be more effective than single-mode interventions (71). The implementation of SDM in clinical practice must be included in the context of the healthcare system where external factors mainly hinder it: time, continuity of care, workflow, and healthcare setting characteristics (70). SDM: KOMPASS is a tool designed to support the implementation of SDM in clinical practice. It functions as a self-assessment instrument tailored for healthcare professionals and managers. Although it still requires validation in real world settings, the tool is comprehensive and structured around five levels: Initiation, Operationalization, Implementation, Integration, and Sustainability, applied to key domains of SDM application: management, organizational culture, education and qualifications, and clinical practice (72). The knowledge about this attitude among HCPs varies in the exact environment; medical students who were at least 18 years old were surveyed about their awareness of SDM approach. The mean SDM knowledge score was 83.6%. Attitudes toward SDM were positive, and a willingness to use this approach seemed to be higher than in previous generations of physicians. Additionally, 74.7% of students have had prior SDM training, and 82.8% of them were interested in learning more about SDM (73). The promotion of SDM encompasses creating training programs for asthma care providers, providing anti-discrimination campaigns, and usage of telemedicine with proper implementation by involving empathy and adjusted treatment therapy.

Structured written treatment plans (WTPs) should serve as a foundation for SDM, as they enhance patient autonomy and improve understanding of asthma and its management. According to pulmonologists, the use of WTPs facilitates better communication with patients and strengthens their self-management skills (10). In pediatric care, WTPs have been associated with reduced missed doses of controller medication, decreased frequency of exacerbations, and improved medication adherence. Addictionally, children, whose parents used WTPs, experienced fewer missed school days, lower reliance on rescue medications, and improvements in Asthma Control Test (ACT) scores. From the caregiver perspective, WTPs increased confidence in managing their child’s exacerbations compared to relying solely on verbal instructions. Nonetheless, due to limited study groups in existing studies, these findings may not be relevant on the larger scale, particularly for CALD patients (74). Asthma patients prefer written, short and precise materials about Maintenance and Reliever Therapy (MART) in the form of leaflets. These leaflets should have information about the description of the effect and usage of the MART approach. Patients also report that they would like to have written forms to better remember important information. Additionally, it has been suggested that such resources could be used to help patients prepare for their consultation with a doctor, yet there is a need for more data especially from HCPs perspective (75). Written asthma action plans (WAAP) were deemed acceptable and preferred by both parties as a way to introduce asthma management into practice. They offer a structured framework that improves treatment awareness and strengthens the therapeutic relationship between patients and healthcare providers. Unfortunately, specialists are also enumerating disadvantages of WAAP like limited literacy skills of patients, difficulty with understanding the WTP, language barriers and forgetfulness about plans (10). Nevertheless, conventional WTPs may present challenges for individuals with low literacy. For example, illiterate Turkish women experienced fewer emergency visits and asthma-related complications, as well as improved disease control, when provided with pictorial treatment plans (76). Similar findings were reported among patients in Malaysia (77). While these studies involved relatively small sample sizes and required replication in more diverse populations, pictorial plans should be considered, particularly for patients from low- and middle-income countries, as a potentially effective and inclusive tool to support asthma self-management.

The absence of sufficient cultural mediators significantly hinders equitable access to healthcare services and the delivery of appropriate treatment. As described earlier, the language barrier is a crucial obstacle in asthma treatment. For example, among patients with asthma from Pakistani and Bangladeshi immigrant communities, friends frequently acted as interpreters, while others were forced to rely on gestures and non-verbal communication to interact with HCPs (78). Consequently, there is a clear need to develop multilingual educational resources to support communication with non-native speakers. Non-profit organizations, such as “Asthma Australia,” have taken steps in this direction by offering asthma information in multiple community languages (79). Physicians engaged in SDM should actively utilize culturally and linguistically appropriate educational materials, as doing so can improve disease awareness and understanding among patients from various ethnic groups. There is also a need to conduct widespread anti-discrimination campaigns among asthma specialists. Asthma risk has been found to increase among Mexican American, Latino and African American individuals who report experiencing high levels of perceived racial discrimination (80). Anti-discrimination interventions could raise awareness of provider bias and ensure egalitarian purposes for care delivery, guaranteeing unprejudiced shared decision making. A review from 2022 summarized interventions which counter racism and unconscious bias in health care. These included dialogues on race and bias, workshops incorporating lectures, and competency training. Most studies (68%) involved medical students as the study population, and both classroom-based and online formats were used to promote anti-discrimination attitudes. Unfortunately, the majority of publications did not have a control group (72%) and none of them used clinical settings. In addition, these studies did not include primarily asthma specialist groups (81, 82).

Similarly, patients with intellectual disabilities require personalized asthma management plans. Davis (83) outlines four key components that should be considered when treating this population: accurate diagnosis based on the patient’s level of cooperation, individualized selection of inhaler devices, instruction in proper inhaler technique, and the use of appropriate communication strategies (83). There is a clear need for dedicated educational materials and training programs tailored to the cognitive and functional capacities of patients with intellectual disabilities. In a small study evaluating inhaler technique and the effectiveness of training interventions, the poorest outcomes were observed in pressurized metered-dose inhalers (pMDIs). While some patients were able to learn proper inhaler use, the study highlighted that poor dexterity and cognitive impairments often led to ineffective inhalation techniques. In such cases, SDM should take into account the patient’s intelligence quotient (IG) and adapt the treatment plan accordingly. Nevertheless, the authors suggest that a more paternalistic approach to care may sometimes be necessary for patients with intellectual disabilities (84). Accurate promotion of SDM in the group of patients with disabilities demands consulting the patient caretaker to assess their ability to manage the asthma treatment of their charge.

Tailored education and training programs can enhance healthcare professionals’ capacity to express empathy. A review based on randomized controlled trials demonstrated that such interventions positively influenced empathy scores among students. These programs equip professionals with the skills necessary to connect with patients on a deeper emotional level, thereby improving communication, fostering trust, and increasing overall patient satisfaction (85).

A more contemporary approach to supporting SDM is the use of telemedicine, whose relevance has grown significantly since the onset of the COVID-19 pandemic (86). Telemedicine is a promising main resolution of transport-related social exclusion for asthma patients. Among patients with asthma, asynchronous visits are used, during which individuals report their symptoms via online portals. Reminder and alert systems are also frequently employed to support disease management T (87). A meta-analysis reviewing the effectiveness of virtual consultations and asthma education found that patients utilizing telemedicine experienced better asthma control and a greater number of symptom-free days (SFDs). This improvement was likely associated with enhanced adherence to inhaled corticosteroids, and among pediatric patients, improved inhaler technique was also observed. Nonetheless, no significant reduction was noted in emergency department (ED) visits or hospitalization rates (86). The Telemedicine Enhance Asthma Management (TEAM-ED) intervention during admissions to the ED, was shown to increase follow-up visits, use of preventive medications, and the mean number of SFDs. Nevertheless, TEAM-ED had no measurable effect on overall medication adherence or asthma morbidity during acute ED visits (88). Another promising approach includes school-based telehealth (SBTH) programs, which aim to support asthma control among pediatric populations. The typical SBTH involves a multidisciplinary team which includes nurses, school workers, and clinicians. The primary aims of SBTH programs include reducing healthcare utilization, minimizing work absenteeism among caregivers, and decreasing school absenteeism among children, while also offering opportunities for remote patient education. These programs employ a long-term care model that enables remote consultations from the school environment, which can be especially beneficial in rural areas where access to comprehensive asthma management programs is limited. Nevertheless, several barriers hinder the implementation of SBTH programs, including limited family engagement and competing responsibilities faced by nurses (89). Broader challenges in telemedicine implementation are often related to issues of financial sustainability, limited access to high-speed internet, particularly in low-income countries, and the need for software licensing (87, 90). Moreover, the cost-effectiveness of telemedicine remains debated (87). Technological exclusion represents another substantial obstacle, especially among older patients who may lack familiarity with digital tools (86). Proper training in telemedicine is essential for HCPs, but it incurs additional costs and requires dedicated time. Furthermore, providers must be prepared to manage technical issues that may arise during consultations, potentially compromising the experience for both clinicians and patients (90). A notable limitation of telemedicine remains the inability to perform a complete physical examination (86). Despite these challenges, the potential benefits of telemedicine warrants its consideration, particularly given the expanded opportunities it offers for enhancing access and facilitation of patient education. Extensive Care System (ECS) integrates continuous, unobtrusive health monitoring (e.g., HRV tracking) with advanced data analytics and real-time feedback via mobile technologies. Grounded in evolutionary science and attachment theory, the ECS emphasizes personalized, predictive, and participatory care, aiming to shift focus from reactive crisis management to proactive wellness support. It draws on established components from health psychology, epidemiology, and learning theory, reinforcing positive behaviors and enabling early intervention through intelligent triage and decision support. While not yet widely implemented, elements of the ECS, such as HRV-based triage and mHealth tools, have shown promising results in pilot studies and reviews, suggesting its potential for real-world application (91).

**FIGURE 1:**
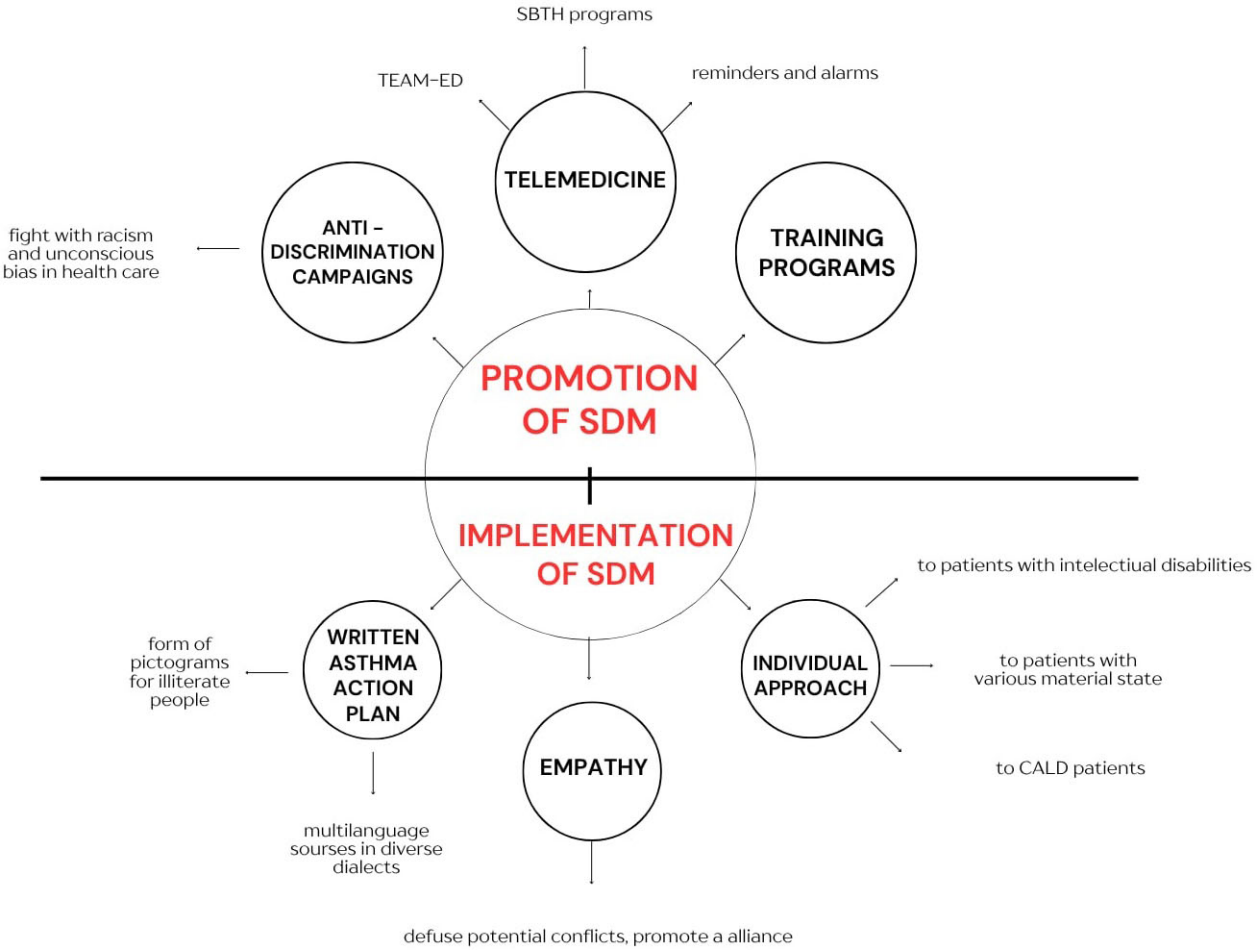
The form of promotion and implementation of SDM in asthma patients.

All aforementioned studies suggest that the proper implementation and promotion of the SDM process is crucial for the effective treatment of patients with asthma. Promotion may involve introducing new IT services such as telemedicine, running and promoting anti-discrimination campaigns aimed at combating inequalities, as well as special training programs for HCPs. From the point of implementation, the main strategies focus on an individualized approach to each patient, tailored to their backgrounds, socioeconomic status, disabilities, and intellectual level. Authors also highlight the importance of written asthma action plans, which serve as an informal agreement between physician and patient, stabilizing the SDM approach. Nevertheless, it is important to note that some of the cited studies were limited to small or highly specific patient populations and healthcare systems. Therefore, their findings should be interpreted with caution and further validated in diverse clinical contexts to avoid overgeneralization and unwarranted extrapolation. Moreover, certain proposed solutions may not be feasible to implement due to structural limitations of healthcare systems or infrastructural constraints–such as restricted access to telemedicine. These aspects warrant thorough investigation to assess their practical applicability across different settings. Teaching and promotion of empathy among healthcare personnel is also a crucial element of SDM strategy (Figure 1).

## 5 Discussion

Shared Decision Making was mentioned for the first time, seemingly in 1972, in an article “Models for Ethical Medicine in a Revolutionary Age” by Robert M. Veatch (92). In 1977 and 1984, Jay Katz used it in his critique of informed consent. SDM started to become a more widely recognized term, and in 2001, the first SDM conference in Oxford was held. It is also possible that at that time the first models were presented (93).

According to the article “Four Models of Physician-Patient Relationship” from 1992, there are four main approaches for how a patient should be involved in medical decision-making: paternalistic (or parental/priestly), informative (or scientific/engineering/consumer), interpretive, and deliberative (94). The choice of what model of relationship to perform is not obvious. While about 58% of patients prefer SDM, still 26% want a paternalistic approach, and 16% want to decide for themselves (95). Some patients who are neutral about their participation in decision making may shift responsibility when faced with a decision they feel is beyond their understanding (12). Among allergologists in the US, approximately 98% are aware of the SDM approach, however, they report implementing it with only 44% of their patients. In contrast, only 22% of patients report perceiving elements of SDM during their clinical encounters (96). There is no single ideal approach to asthma management, as patients’ experiences vary and influence their preferences.

In preparing this article, we conducted a narrative review of the literature using databases such as PubMed, Google Scholar, and Elsevier. We primarily included studies published between 2015 and 2025, however, to illustrate historical shifts in the development of SDM or in cases where more recent data were lacking, we also referred to earlier publications. The oldest source cited in this review dates to 1992. Based on our work we have decided to create a model adjusted to the needs of asthmatic patients. To create a theoretical model, we have gathered elements of other existing models of SDM prevalent in medicine, and chose those that answer the problems mentioned by articles focused on barriers asthmatic patients face in achievement of adequate care. The Empathy-Centered Shared Decision-Making Model is aimed to emphasize the creation of a supportive environment that fosters empathy and trust. It integrates various elements to ensure effective communication, accurate diagnosis, and personalized treatment plans, however it still needs evaluation

### 5.1 Create a supportive environment

The first step involves establishing a clinical environment that prioritizes empathy and trust. This element was chosen based on The Four Habits Model, as it answers patients’ needs for empathic communication for a successful consultation process (44). Anxiety and self-efficacy, as well as anxiety and sleep quality, act as chain-mediated factors linking empathy to serum IL-6 levels. Specifically, empathy reduces patient anxiety, which in turn directly enhances self-efficacy and sleep quality, both of which influence IL-6 concentrations. Anxiety negatively impacts self-efficacy by acting as a psychological barrier to proactive behavior and positive health management (43). Physiological indicators like IL-6 levels and sleep quality can be integrated into the model design by focusing on empathy and its psychological mediators, promoting better biological outcomes through increased patient engagement and improved emotional well-being. HCPs should demonstrate genuine concern for the patient’s well-being, actively listen to their concerns, and validate their experiences. This can be achieved through active listening, such as summarizing what the patient has said and asking follow-up questions to show understanding. Elements of non-verbal communication like body language and facial expressions should convey openness, helping to build rapport (66).

### 5.2 Assessment of situation

According to the Purposeful SDM framework, the decision to apply SDM should begin with an assessment of the individual context in which the patient presents. For patients with asthma, this context may be shaped by factors such as disease severity. Severe asthma affects approximately 9,5% of patients (97), and these individuals may require a different consultation strategy than those with milder forms of the disease. Additionally, cultural background and language barriers can significantly impact the consultation process, leading to misunderstanding (24, 25) or difficulties in understanding proposed treatment plan (27). Comorbidities, particularly mental health conditions, also play a critical role (18). This factor, highlighted as well in the MIND-IT model, should be carefully considered to enable a meaningful and satisfactory consultation process.

### 5.3 Collaborative discussion

Discussion is the most important element of SDM, prevalent in all of the models. HCPs should facilitate a collaborative discussion about treatment options, ensuring a patient’s feeling of involvement in the decision-making process. This can be achieved through clear outlining of the available treatment options, their benefits and potential side effects, and inviting patients to ask questions and express concerns.

### 5.4 Education

Lack of adequate education on illness and treatment is a barrier commonly mentioned in the studies on asthma patients’ needs (31, 32, 33). Models like Longitudinal SDM provide an interesting solution to that problem. To enhance understanding HCPs should offer educational materials tailored to the patient’s literacy level and language preferences. This can include written materials and visual aids, like diagrams or pictorial representation to illustrate inhaler techniques and medication usage (10, 74, 75).

Through the whole medical encounter PtDAs can play a crucial role by facilitating informed choice. Patients provided with information about their treatment options in a structured format, may find it easier to weigh the pros and cons. Moreover, encouraging them to reflect on their values and preferences, may lead to more personalized treatment decisions. Similarly to IP-SDM, HCPs should consider referring patients to specialists for further evaluation and management. According to The Severe Asthma Charter from 2018, patients expect accessibility of other specialist consultation if needed, as asthma is a chronic illness, with exacerbations commonly coexisting with other comorbidities (32). Electronic apps and Artificial Intelligence (AI) techniques are a promising route for the future of asthma treatment. A review that included studies on use of AI for SDM in primary care for patients with diabetes and stroke survivors and secondary care for patients who needed treatment for their knee or neonatal care, suggests that AI can present personalized treatment options, with risks and benefits. This can be used to educate patients, as AI models are able to process all information on patients’ care and create evidence based recommendations. This may open more time for HPCs during visits to focus solely on the patient. Nevertheless, implementation in practice may be challenging, due to lack of trust and inability to understand the process behind decision making of AI. There is a need for more research on AI tools, implementation, especially for asthma treatment (98). To facilitate systemic adoption, SDM practices, such as structured asthma action plans or patient preference checklists, should be embedded within electronic health record systems and quality benchmarks.

The assessment of SDM remains challenging due to the lack of standardized and universally accepted evaluation tools. Since there are numerous factors that impact treatment outcomes, direct measurement of SDM impact is difficult (99). Patient feedback remains a valuable proxy for evaluating SDM quality, however, the impact of SDM should go beyond patient satisfaction to include concrete clinical endpoints such as asthma control, frequency of exacerbations, and healthcare resource utilization.

Shared Decision Making can be applied across a range of clinical scenarios in asthma management. The considerable diversity in medication types, routes and frequency of administration, as well as the presence of comorbidities in asthma, underscores the importance of implementing shared decision-making (SDM) in clinical care. SDM increases the likelihood of developing a treatment plan that aligns with the patient’s preferences, needs, and circumstances, thereby enhancing treatment satisfaction and adherence (35, 36). This approach is particularly relevant for athletes with asthma, for whom treatment plans should be carefully tailored to meet their specific needs and to ensure that the condition does not hinder their ability to achieve performance goals. Such plans should be reviewed and adjusted regularly in accordance with the patient’s current health status and activity levels. For example, individuals with low symptom awareness may benefit from the regular use of a peak flow meter to support better self-monitoring and disease management (100). Another group that can benefit from SDM, are patients with poor-controlled asthma. Implementation of SDM prolongs time without exacerbation among children (101).

For instance, among patients with osteoarthritis, those who engaged in better-informed decision-making reported slightly improved health outcomes and less decisional regret (102). In stroke care, decision aids improved patients’ knowledge but did not show a clear link to increased adherence to anticoagulants (103). Conversely, in dermatology patients prescribed topical corticosteroids who received thorough counseling were less apprehensive about side effects and more likely to adhere to treatment, indicating that SDM can influence both satisfaction and adherence (104). For asthma patients SDM proves to improve their life quality and adherence. Those individuals who report higher participation in SDM tend to have better satisfaction with their medication choice (12). There is also interest in SDM’s potential to reduce healthcare costs by promoting appropriate resource use and avoiding unnecessary intervention, but the relationship has not yet been conclusively demonstrated (105).

Shared Decision Making models must be adapted to local healthcare contexts, taking into account technological infrastructure, cultural beliefs, and socioeconomic disparities to ensure equitable implementation.

Despite SDM being the currently preferred model of asthma management, the research on the matter is not sufficient. There is still a need for research on the applicability of different models, standardizing evaluation tools, and more in depth studies on interventions focused on answering the needs of patients from minority groups.

## 6 Conclusion

Asthma is a chronic obstructive inflammatory pulmonary disease where the environmental framework influences the course of this disease. The SDM concept, where both patients and their HCPs are engaged in the therapeutic process, is considered an important factor in proper asthma management. HCPs must consider all external and internal agents like patients’ backgrounds, disabilities and health awareness influencing patient-healthcare relations. A standardized tool to evaluate SDM has not yet been developed, but important elements have been presented: education on advantages and disadvantages of different treatments, discussion of patients’ preferences and needs and ensuring comprehension of asthma management. The presented models show various schemes of SDM approach, underlining cooperative therapeutic decisions. The shared decision-making principles require widespread promotion among medical society by introducing training programs or anti-discrimination campaigns, as well as the main strategies for implementing SDM approach focusing on individual attitudes toward each patient. Telehealth is a novel solution for implementation of SDM and fighting against the distance barrier problem, and should be propagated further.

## 7 Summary

Asthma is a chronic inflammatory disease characterized by a history of respiratory symptoms, including wheezing, shortness of breath, chest tightness, and cough. Inhaled corticosteroids (ICS) and long-acting beta-agonists (LABA) are the first-line treatments, improving disease control; however, regular administration is essential for disease control and prevention of exacerbations. Medication adherence in this regard varies and remains suboptimal. According to the World Health Organization (WHO), the effectiveness and quality of the healthcare system constitute one of the five key dimensions influencing medication adherence, with the patient-physician relationship playing a significant role. Shared Decision-Making (SDM) is a concept that involves both the patient and their healthcare provider (HCP) in the decision-making process, ensuring that patients receive comprehensive information on available treatment options and associated risks so that the chosen therapy aligns with their expectations. Although the term emerged in the 1980s and various models have been tested in medical practice over the years, no standardized or well-validated model has been specifically recommended for asthma management. Furthermore, no standardized tool exists for evaluating SDM in this context. Current asthma guidelines incorporate SDM in decisions regarding inhaler use, and HCPs are encouraged to implement SDM approaches, as they are associated with improved disease control. This review aims to analyze and synthesize recent findings from the medical literature regarding the implementation and evaluation of SDM models in clinical practice, with particular emphasis on factors most critical for enhancing asthma treatment efficacy by strengthening the patient-physician relationship and improving medication adherence.

Patient-centered interventions can be analyzed from both the patient and HCPs perspectives. Patients’ engagement in SDM varies, ranging from active participation in treatment decisions to complete reliance on the expertise of their healthcare providers. Individuals with asthma often express the need for a more comprehensive explanation of their condition and the rationale behind treatment choices. They emphasize the importance of effective verbal and non-verbal communication, as well as cultural competence among HCPs, as critical factors in asthma care. Conversely, HCPs cite time constraints and insufficient patient education as key barriers to the full implementation of asthma treatment strategies. Additionally, various challenges must be considered, including patients’ health beliefs, limited health literacy, conflicts with healthcare providers, lower socioeconomic status, and household income, all of which can hinder SDM and disrupt continuity of care.

There are many models of SDM that provide a generalized plan and advice to efficiently introduce those important elements into practice. Most of them focus on educating patients on pros and cons of different treatments, discussing patients preferences and needs, and at the end receiving confirmation, if the patient understands instructions. Better comprehension of strengths and limitations of those models can help choose the best way of conducting visits to ensure optimal outcomes for patients. Some models like three-talk or SHARE, focus on simplifying communication and ensuring patient participation, while others like Six Steps of SDM provide a structured framework for engaging patients, or like IP-SDM highlight the importance of interdisciplinary collaboration. There are also models like Purposeful SDM that emphasize tailoring models in specific medical scenarios. Combination of elements from those approaches may be helpful if implemented in practice by an allergist. The Severe Asthma Charter was developed in consultation with patients and underscores the importance of patient involvement in treatment decisions, positioning SDM as a fundamental component of asthma care. A key principle outlined in the Charter is that patients seek comprehensive education about their disease. Education is a central element in most SDM models, and in some–such as the Longitudinal-SDM and MIND-IT models–it serves as the primary objective, aiming to facilitate informed decision-making. These models utilize Patient Decision Aids (PtDAs), such as educational materials, to enhance patient understanding and engagement in the decision-making process. Beyond education, establishing trust and fostering a strong patient-provider relationship is essential for effective SDM. Empathy plays a critical role in this dynamic and is a core component of The Four Habits model, which provides structured guidance on when and how to express empathy both verbally and non-verbally during clinical encounters. Evidence suggests that empathetic interactions with HCPs can yield measurable physiological and psychological benefits; for example, studies demonstrated that patients who experienced empathy during consultations exhibited lower serum IL-6 levels, improved sleep quality, and greater self-efficacy in managing their treatment. A primary concern for patients with asthma is the extent to which their condition limits daily life. The impact of treatment on quality of life is a crucial consideration in SDM, as highlighted by the 3-Circle Conceptual Model, which illustrates how various contextual factors–such as family dynamics, medical circumstances, and evolving personal needs–interact within the DM process. This model is further supported by the Multistep SDM Pathway, designed to facilitate the integration of such contextual factors into clinical practice. Another principle emphasized in the Severe Asthma Charter is the need for timely and direct referrals to appropriate specialists based on disease severity. The Interprofessional SDM model addresses this by promoting a collaborative approach to decision-making involving the patient, their primary HCP, and at least one additional specialist. To further refine SDM strategies in varying clinical contexts, the Purposeful-SDM model was developed, offering a structured approach to navigating different decision-making scenarios. Additionally, the SDM: KOMPASS tool was developed to enable self-assessment for HCPs and healthcare administrators; however, it has yet to be validated on a large scale. The most common element of those methods is introducing patients to treatment options and education on their illness, as it proves important to patients that suffer from allergy or asthma. Many of them can also benefit from more involvement of other specialists.

The standardized tool to evaluate SDM has not yet emerged, as it still does not have clear terminology nor accreditation standards and guidelines, but it seems that implementing SDM into practice may improve feelings of autonomy, competence and relatedness of patients. On the other hand there are mixed results on impact on adherence. The effective implementation of SDM in medical practice requires the development of various interventions to strengthen the patient-HCP relationship, improve communication, and build trust. While some SDM models provide broad conceptual frameworks for integrating SDM into clinical practice–such as the Three-Talk Model–others adopt more detailed, multi-step methodologies. Researchers continue to develop practical tools to enhance SDM implementation in routine care. The ADAPT-NC Toolkit was specifically designed to support physicians in applying SDM principles, while the Share to Care intervention program was established to facilitate the introduction of SDM into healthcare systems. Telemedicine offers significant opportunities to enhance asthma management, particularly among younger and well-educated patients, leading to better symptom control and more symptom-free days. However, it also poses challenges in establishing trust, especially with new patients. Empathy from healthcare providers and continuity of care play a crucial role in improving patient outcomes. Information and Communication Technologies (ICTs) utilizing the use of electronic health care tools (eHealth), such as health mobile apps (for example Coach McLungsSM), can aid in patient education, with preferences influenced by social and educational background. Many patients favor structured, written treatment plans, while those with lower literacy levels benefit from visual aids, such as pictograms, to enhance understanding and adherence.

Shared Decision Making could be a significant aspect of asthma treatment, with HCPs considering individual, family, and institutional factors when making therapeutic decisions. Various SDM models have been discussed in this review, highlighting both their strengths and limitations, as well as their applicability in asthma treatment. Most of them focus on educating patients on pros and cons of different treatments, discussing patients’ preferences and needs, and at the end receiving confirmation, if the patient understands instructions. There is a clear need for the development of interventions to more effectively integrate SDM into clinical practice. While progress has been made, there are still opportunities to enhance HCPs’ ability to create a supportive environment for patients. A particular gap exists in addressing the needs of patients from lower socio-economic backgrounds, with limited literacy, access to new technologies, or from diverse racial and cultural backgrounds. These groups are more likely to face barriers in accessing education, treatment options, and communication with their healthcare providers.
