# Supplementary material for: Shared decision making - a review of its evaluation, efficacy, and applicability in asthma
Source: Front Med (Lausanne). 2025 Aug 12;12:1639805. doi: 10.3389/fmed.2025.1639805 (PMC12378715; doi:10.3389/fmed.2025.1639805)

# *Supplementary Material*

## 1. ***Supplementary table 2*** Tab. 2 The comparison of SDM models

| Element | Charles et al. | The Three Talk model | IP-SDM | The SHARE model | MIND-IT | 3-Circle Conceptual Model and Multistep Shared Decision-Making Pathway | Purposeful SDM | Longitudinal SDM | The Six Steps of SDM | The Four Habits |
|---|---|---|---|---|---|---|---|---|---|---|
| Key feature | General description of most important elements of SDM | A simplified structure of key components of a SDM conversation | Emphasizing the importance of interprofessional collaboration | Detailed breakdown of shared decision-making steps relevant to the clinical encounter | Recognizing the impact of patient individuality on the quality and outcomes of SDM processes | Recognizing the impact of patient individuality on the quality and outcomes of SDM processes | Emphasizing the need to tailor the approach to the specific clinical situation | Utilizing educational resources to enhance patient knowledge and engagement | Detailed breakdown of shared decision-making steps relevant to the clinical encounter | A detailed account of strategies to foster a supportive and trusting atmosphere in clinical encounters |
| What asthma patients' problem does it answer? | -The need for shared involvement in decision-making | -The need for shared involvement in decision-making | -The need for shared involvement in decision-making | -The need for shared involvement in decision-making | -The need for shared involvement in decision-making<br>-The need for education<br>-The importance of incorporating personal life context | -The need for shared involvement in decision-making | -Highlighting the variety of clinical scenarios | -The need for education | -The need for shared involvement in decision-making | -The need for empathy and trust |

| | | | | | | | | | | |
|---|---|---|---|---|---|---|---|---|---|---|
| Sources | (Charles et al., 1997) | (Elwyn et al., 2017) | (Légaré et al., 2011) | (Hargraves et al., 2020) | (Barradell et al., 2023) | (Rennke et al., 2017) | (Hargraves et al., 2019) | (LoBrutto et al., 2022) | (Clayman et al., 2023) | (Frankel & Stein, 2001) |

***2. Supplementary figure 1*** Figure 1. The form of promotion and implementation of SDM in asthma patients.

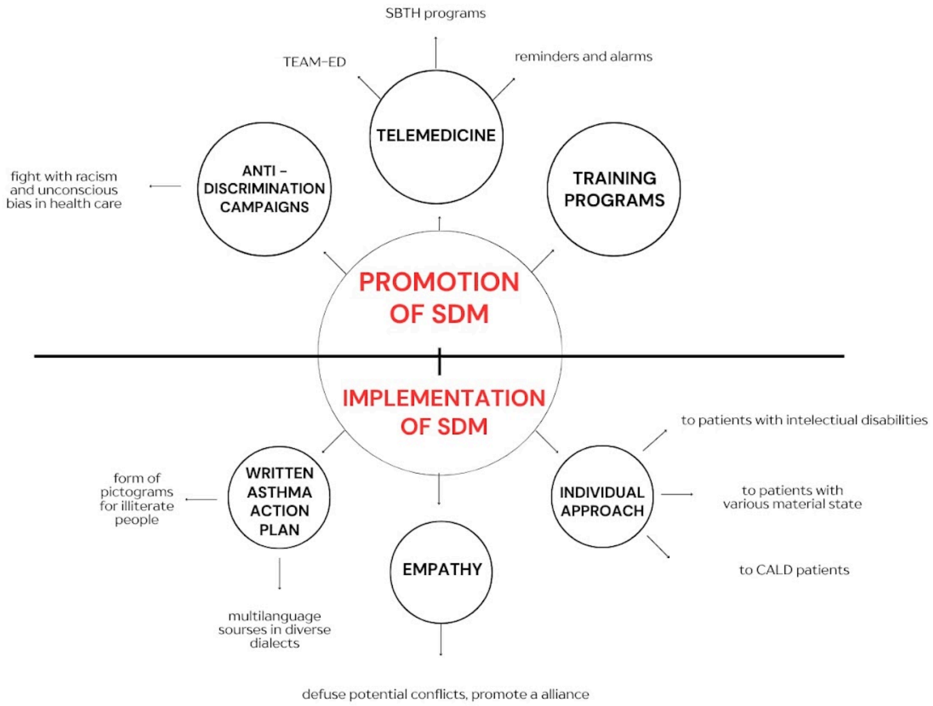

Supplement: Supplementary file 1 [file Data_Sheet_1.pdf]
